# L-shaped association of thiamine intake and risk for peripheral artery disease in US adults: a cross-sectional study

**DOI:** 10.3389/fnut.2024.1437930

**Published:** 2024-10-01

**Authors:** Zhiyong Dong, Qingyun Wang

**Affiliations:** Department of Cardiothoracic Surgery, Beijing Shunyi Hospital, Beijing, China

**Keywords:** peripheral artery disease, thiamine intake, a cross-sectional study, L-shaped association, US adults

## Abstract

**Background:**

The relationship between thiamine intake and risk for peripheral artery disease (PAD) is unknown. We aimed to clarify the role of thiamine intake on risk for PAD and the implications of this relationship. The secondary objective of this study is to explore the potential non-linear dose–response relationship between exposure to thiamine intake and outcome risk for PAD.

**Methods:**

We conducted a cross-sectional study involving 6,112 participants with US adults from the National Health and Nutrition Examination Survey (1999–2004). Logistic regression and restricted cubic spline were utilized to substantiate the research objectives.

**Results:**

The overall prevalence of risk for PAD was 7.9, 51% in males and 49% in females. After multivariable adjustment, lower thiamine intake was significantly and nonlinearly associated with higher risks of PAD among participants. Furthermore, we discovered L-shaped associations (*p* = 0.082) between thiamine intake and the risk of PAD, with an inflection point at 0.66 mg/day. Accordingly, in the threshold effect analysis, there was an inverse association between dietary thiamine intake and the risk in participants with dietary thiamine intake <0.65 mg/day. Compared to participants with thiamine intake below the inflection points, those with higher levels had a 31% lower risk for PAD (OR, 0.69; 95% CI: 0.51, 0.95). Further subgroup analysis showed no significant interactions between the subgroups (all *p* values for interaction were > 0.05).

**Conclusion:**

A non-linear association was revealed, showing that low and high levels of thiamine intake were associated with an increased the risk of peripheral artery disease in American adults. The inflection point at 0.66 mg/day and lower risk of PAD at 0.65–1.13mg/day of dietary thiamine intake may represent intervention targets for lowering the risk of PAD. The findings of this study require further validation and confirmation.

## 1 Introduction

Peripheral artery disease (PAD) is a prevalent and debilitating condition worldwide, characterized by intermittent claudication and critical limb ischemia. It continues to be a global burden, affecting approximately 12 to 20% of Americans 60 years and older (1). Moreover, PAD affects lower limb activity, may lead to lower limb ischemic ulcers and amputations, and also significantly increases the risk of cardiovascular and cerebrovascular events and death in patients (2), resulting in a severe economic burden (3). Therefore, it is crucial to identify more modifiable risk factors of PAD in diets and lifestyles. In 2014, Naqvi et al. (4) reported that the prevalence of PAD was negatively associated with the intake of fiber, folate, and vitamins A, B6, C, and E after adjusting for age, sex, hypertension, diabetes, and smoking. However, there is little data about the association between thiamine and PAD.

Thiamine, also called vitamin B1, plays a crucial role in various metabolic processes within the human body (5–7). It is an essential water-soluble vitamin that cannot be synthesized by the human body and might be corrected by exogenous thiamine and benfotiamine (8–10). Thiamine is primarily absorbed in the jejunum of the small intestine through active transport mechanisms involving thiamine transporters (THTR-1 and THTR-2) (11–13). These transporters facilitate the uptake of thiamine in a sodium- and ATP-dependent manner (14). Additionally, while gut bacteria in the large intestine can synthesize thiamine (15), this contribution to overall thiamine status in the body is considered minimal due to limited absorption from the colon (16, 17). Thiamine is involved in processes such as oxygen transport, energy metabolism, enzyme function, and DNA synthesis (6, 7). However, thiamine deficiency causes lactate accumulation, reducing peripheral vascular resistance (18, 19). Patients with wet beriberi have cardiovascular symptoms, which manifest as high-output cardiac failure, systemic vasodilatation, lactic acidosis, edema, and fluid retention (20). Currently, there is insufficient evidence regarding the relationship between thiamine intake and the risk for PAD. Understanding the relationship between thiamine intake and the risk for PAD is of critical importance in the fields of health and public health.

Based on the studies above, we hypothesize that thiamine may serve as an essential indicator of the risk for PAD. The primary aim of this study is to demonstrate the correlation between thiamine intake and the risk for PAD. Additionally, we seek to explore the potential non-linear dose–response relationship between thiamine and the risk for PAD. To achieve these objectives, we conducted a cross-sectional study involving 6,112 adult participants in the United States.

## 2 Materials and methods

### 2.1 Study design and population

Our current analyses were based on the National Health and Nutrition Examination Survey (NHANES) conducted by the National Center for Health Statistics (NCHS). The NHANES protocols were approved by the NCHS Research Ethics Review Board. NHANES obtained informed written consent from all participants. The study design, protocols, and datasets are available on the NHANES website.^1^

Our study included three cycles of NHANES data from 1999 to 2004 (*n* = 31,126). The data for each cycle contains five parts: demographics, dietary data, examination data, laboratory data, and questionnaire data. Participants with a valid ankle-brachial index (ABI ≤ 1.4) and dietary intake were found in examination data and dietary data (*n* = 6,749, aged 40 years or older). Then, we excluded participants with a lack of other covariates information (*n* = 637). Finally, A total of 6,112 participants were included in the final analysis.

### 2.2 Exposure

In the NHANES, the dietary intake data were used to estimate the types and amounts of foods and beverages consumed during the 24 h period before the interview (midnight to midnight) and to estimate intakes of energy, nutrients, and other food components from those foods and beverages. Following the dietary recall, respondents were asked questions on water consumption during the previous 24 h period, salt use, and whether the person’s intake on the last day was usual or unusual. All dietary interviewers had to complete an intensive one-week training course followed by supervised interview practices before working independently in the field to validate the accuracy of 24 h dietary recalls. We obtained an individual thiamine intake from the dietary intake data.

The measurement of ABI was conducted in individuals over 40 years of age. This particular measurement approach has been documented in previous studies. Systolic blood pressures were taken in the right arm (brachial artery) and both ankles (posterior tibial artery) after a brief rest period. If the participant’s readings from the right arm were unavailable due to any circumstances that could affect accurate measurements (e.g., open wounds, dialysis shunts), the left arm was used for the brachial pressure measurement. Subjects aged 40–59 years had their systolic blood pressure measured twice, whereas those aged 60 years and older had it measured only once. The ABI was determined by dividing the ankle’s average systolic blood pressure (ASBP) by the ASBP in the arm. As participants aged 60 and older only had one reading, the first reading represented the mean values. Peripheral arterial disease (PAD) was defined as an ABI of less than 0.9.

### 2.3 Covariates

Standardized questionnaires collected information on age, gender, race/ethnicity, education levels, family income, smoking status, and disease status (history of hypertension, coronary heart disease, diabetes, and tumor). Participants’ body mass index (BMI, kg/m^2^) was measured at a Mobile Examination Center. All details of these variables, including the measurement methods, questionnaire data, and variable list, can be found on the official NHANES website.^2^

Additionally, race/ethnicity was classified into the categories of Mexican American, other Hispanic, non-Hispanic White, non-Hispanic Black, or other races. Education levels were categorized as less than high school, high school or equivalent, college, or above. Family income was classified as low income or high income, based on the family poverty income ratio (PIR), where a PIR of less than 1.30 corresponded to low income and a PIR of 1.3 or greater corresponded to high income. Smoking status was categorized as current smoker, past smoker, or non-smoker.

### 2.4 Statistical analysis

We compared the characteristics of the participants based on quartiles of dietary thiamine intake (<0.96, 0.96–<1.36, 1.36–<1.88, ≥1.88 mg/day). Proportions were used for categorical variables, while means ± standard deviations (SDs) were used for continuous variables. Categorical variables were compared using chi-square tests, while continuous variables were compared using analysis of variance (ANOVA). The association between dietary thiamine intake and the risk of PAD was assessed using binary logistic regression models, adjusting for significant covariates. These covariates were selected based on clinical interest and previous scientific literature. Three models were constructed: Model 1 had no adjustments, Model 2 was additionally adjusted for age, race, sex, and PIR, and Model 3 was additionally adjusted for all covariates.

A restricted cubic spline model was utilized to examine potential non-linear dose–response associations between dietary thiamine intake and the risk of PAD. Dietary thiamine intake was treated as a continuous variable with four knots (5th, 35th, 65th, and 95th percentiles), as suggested by Harrell. Non-linearity was tested by including a quadratic term in the regression models. If a non-linear correlation was detected, a two-piecewise regression model was employed to determine the threshold effect of dietary thiamine intake on the risk of PAD based on the smoothing plot. Pre-specified subgroup analyses were conducted based on subgroup variables. Sensitivity analyses were performed to assess the robustness of the study findings and the impact of various association inference models on the conclusions. The effect sizes and *p*-values from all these models were reported and compared. All analyses were performed using R Statistical Software (Version 4.2.0, http://www.R-project.org, The R Foundation). A two-sided *p*-value of <0.05 was considered statistically significant.

## 3 Results

### 3.1 Study population and characteristic

A total number of 6,112 participants aged 40 years were included in the analysis. Of these, the overall prevalence of risk for peripheral artery disease was 7.9%. The baseline characteristics of the groups stratified by quartiles of dietary thiamine intake are shown in Table 1. The four groups differed in sex, age, PIR, education, literacy, hypertension, smoking, and diabetes mellitus (all *p* values <0.05). Otherwise, the distribution of participants’ characteristics (body mass index, diabetes mellitus, Tumor, and CHD) between quartile groups of dietary thiamine intake was similar (all *p* values>0.05).

**Table 1:** Baseline characteristics of the participants stratified by the dietary thiamine intake quartiles.

Characteristic	Overall, *N* = 6,112^a^	Q1 (<0.96)^a^	Q2 (0.96–1.36)^a^	Q3 (1.36–1.88)^a^	Q4 (>1.88)^a^	*p*-value^b^
Age (years)	60 (13)	60 (13)	61 (13)	60 (13)	56 (13)	<0.001
Sex, male	3,116 (51%)	551 (36%)	656 (43%)	826 (54%)	1,083 (71%)	<0.001
PIR	2.48 (1.61)	2.09 (1.62)	2.32 (1.56)	2.69 (1.58)	2.90 (1.63)	<0.001
BMI, kg/m^2^	27.8 (5.6)	27.9 (5.6)	27.6 (5.7)	28.0 (5.5)	27.8 (5.6)	>0.9
Age. group						<0.001
40–59 years	2,960 (48%)	715 (47%)	690 (45%)	721 (47%)	834 (55%)	
60–79 years	2,559 (42%)	665 (43%)	666 (44%)	657 (43%)	571 (37%)	
80+ years	593 (9.7%)	149 (9.7%)	172 (11%)	152 (9.9%)	120 (7.9%)	
Race						<0.001
Non-Hispanic White	3,364 (55%)	685 (45%)	841 (55%)	917 (60%)	921 (60%)	
Non-Hispanic Black	1,098 (18%)	389 (25%)	273 (18%)	208 (14%)	228 (15%)	
Mexican American	1,271 (21%)	363 (24%)	313 (20%)	305 (20%)	290 (19%)	
Other/multiracial	149 (2.4%)	28 (1.8%)	50 (3.3%)	38 (2.5%)	33 (2.2%)	
Other Hispanic	230 (3.8%)	64 (4.2%)	51 (3.3%)	62 (4.1%)	53 (3.5%)	
Education level						<0.001
Less than high school	1,984 (32%)	635 (42%)	514 (34%)	438 (29%)	397 (26%)	
High School	1,464 (24%)	325 (21%)	376 (25%)	407 (27%)	356 (23%)	
More than high school	2,664 (44%)	569 (37%)	638 (42%)	685 (45%)	772 (51%)	
Income						<0.001
Low income	1,566 (26%)	515 (34%)	387 (25%)	331 (22%)	333 (22%)	
High income	4,546 (74%)	1,014 (66%)	1,141 (75%)	1,199 (78%)	1,192 (78%)	
PAD	483 (7.9%)	142 (9.3%)	130 (8.5%)	116 (7.6%)	95 (6.2%)	0.012
Smoke						0.008
Current smoker	1,181 (19%)	332 (22%)	301 (20%)	258 (17%)	290 (19%)	
Former smoker	2,112 (35%)	478 (31%)	532 (35%)	555 (36%)	547 (36%)	
Never smoker	2,819 (46%)	719 (47%)	695 (45%)	717 (47%)	688 (45%)	
BMI.group						>0.9
Normal (18.5 to <25)	1,599 (26%)	404 (26%)	391 (26%)	404 (26%)	400 (26%)	
Obese (30 or greater)	2,027 (33%)	521 (34%)	509 (33%)	490 (32%)	507 (33%)	
Overweight (25 to <30)	2,427 (40%)	589 (39%)	611 (40%)	620 (41%)	607 (40%)	
Underweight (<18.5)	59 (1.0%)	15 (1.0%)	17 (1.1%)	16 (1.0%)	11 (0.7%)	
Thiamine intake	1.36 (0.84)	0.73 (0.20)	1.15 (0.12)	1.58 (0.15)	2.39 (0.83)	<0.001
CHD	402 (6.6%)	90 (5.9%)	107 (7.0%)	94 (6.1%)	111 (7.3%)	0.3
Hypertension	2,608 (43%)	711 (47%)	690 (45%)	644 (42%)	563 (37%)	<0.001
Tumor	764 (13%)	163 (11%)	191 (13%)	201 (13%)	209 (14%)	0.062
Diabetes	864 (14%)	217 (14%)	229 (15%)	221 (14%)	197 (13%)	0.4

a Median (SD); *n* (%).

b Kruskal-Wallis rank sum test; Pearson’s Chi-squared test.

Participants with higher dietary thiamine intake were younger, more likely to be male, never smokers, and had higher education levels and PIR. More importantly, we observed that participants with a higher thiamine intake tended to have a lower risk of PAD rates (Quartile 1: 9.3%, Quartile 2: 8.5%, Quartile 3: 7.6%, Quartile 4: 6.2%, *p* = 0.012).

### 3.2 Association of dietary thiamine intake with PAD

Table 2 displays the results of the univariate and multivariable logistic regression analysis. We designed 3 logistic regression models to investigate the independent role of thiamine intake in the risk of peripheral artery disease.

**Table 2:** Odds ratios (ORs) and 95% confidence interval (CI) of the thiamine quartiles for PAD.

Characteristic	Model 1			Model 2			Model 3		
	OR^a^	95% CI^a^	*p*-value	OR^a^	95% CI^a^	*p*-value	OR^a^	95% CI^a^	*p*-value
Quantiles									
Q1	Ref			Ref			Ref		
Q2	0.91	0.71, 1.17	0.4	0.90	0.69, 1.17	0.4	0.89	0.68, 1.16	0.4
Q3	0.80	0.62, 1.03	0.090	0.89	0.68, 1.17	0.4	0.96	0.72, 1.26	0.8
Q4	0.65	0.49, 0.85	0.002	0.81	0.61, 1.09	0.2	0.88	0.65, 1.19	0.4

a OR, odds ratio; CI, confidence interval. PAD, peripheral artery disease.

Model 1 adjusted for none. Model 2 adjusted for age, sex, race, and PIR. Model 3 adjusted for all covariates.

When the thiamine intake was treated as a categorical variable based on quartiles and the first quartile was used as a reference, participants in the second to fourth quartile had a lower risk of PAD in all three models. The univariate analysis showed the OR with 95%CI for PAD from lowest to highest thiamine intake were 1.00(reference), 0.91(0.71–1.17), 0.80(0.62–1.03) and 0.65(0.49–0.85), respectively. After multivariate adjustment including age, sex, race, education level, PIR, BMI, smoking status, and hypertension status (Model 3), the multivariate-adjusted ORs and 95% confidence intervals (CIs) were 1.00 (reference), 0.89 (0.68, 1.16), 0.96 (0.72, 1.26), and 0.83 (0.65, 1.19), respectively.

### 3.3 The detection of nonlinear relationships

**Figure 1:**
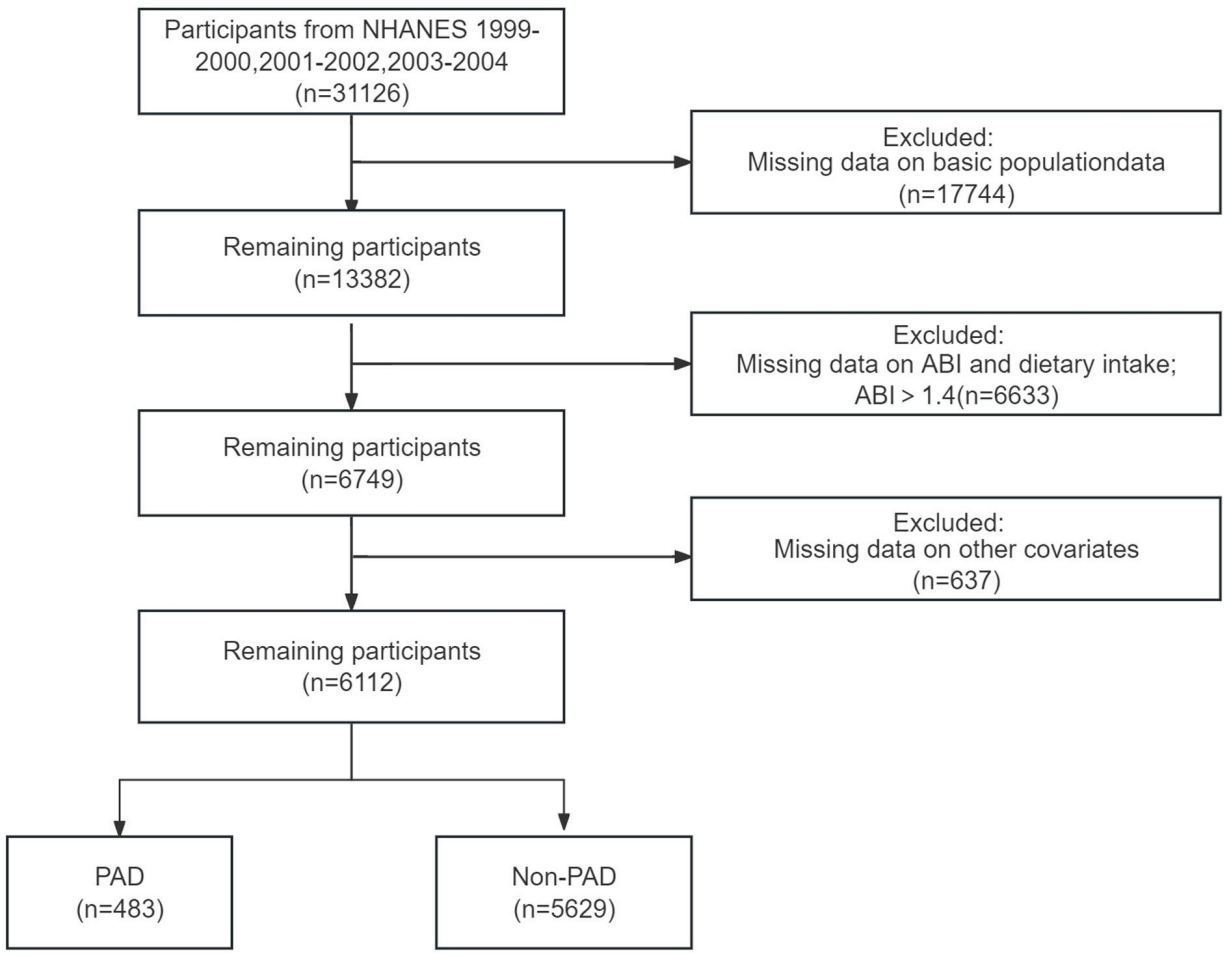
Inclusion and exclusion flowchart of the study.

**Figure 2:**
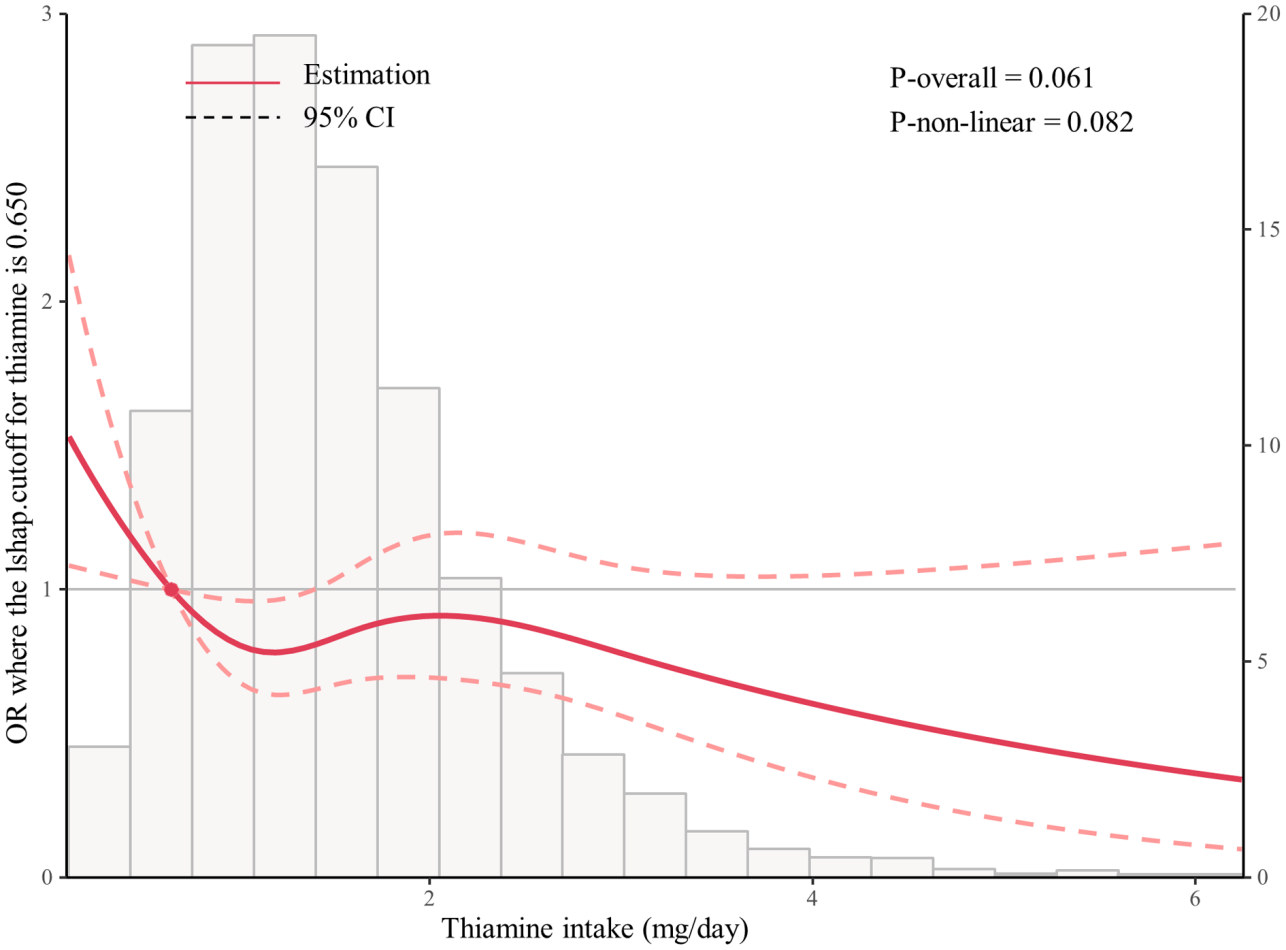
The restricted spline curve shows the relationship between dietary thiamine intake and PAD.

As shown in Figure 1, multivariable-adjusted restricted cubic spline analyses suggested “L-shaped” associations of Thiamine intake with the risk for PAD (*p* for nonlinearity 0.082). The risk for PAD decreased with thiamine intake levels up to the turning point (0.65). In the curve, we found that OR was less than 1. Accordingly, the threshold effect analysis showed that there was an inverse association between dietary thiamine intake (OR, 0.69; 95% CI: 0.51, 0.95) and PAD in participants with dietary thiamine intake ≥0.65 mg/day and those with dietary thiamine intake <0.65 mg/day (Figure 2).

When dietary thiamine intake was assessed as categories quartiles, compared with participants in the second quartiles (0.65–1.13 mg/day, adjusted OR, 0.67; 95% CI, 0.48, 0.95), the risk of PAD was lower not only in participants in the first quartile (<0.65 mg/day; reference) but in those in the third quartile (≥1.13 mg/day; adjusted OR, 0.70; 95% CI,0.51, 0.97) (Table 3).

**Table 3:** Odds ratios (ORs) and 95% confidence interval (CI) of thiamine categories for PAD.

Characteristic	Crude Model			Adjusted Model		
	OR^a^	95% CI^a^	*p*-value	OR^a^	95% CI^a^	*p*-value
Categories						
Q1 (<0.65)	Ref			Ref		
Q2 (0.65–1.13)	0.78	0.57, 1.08	0.13	0.67	0.48, 0.95	0.022
Q3 (≥1.13)	0.66	0.50, 0.89	0.005	0.70	0.51, 0.97	0.030
Threshold						
Q1 (<0.65)	Ref			Ref		
Q2 (≥0.65)	0.70	0.53, 0.93	0.012	0.69	0.51, 0.95	0.018

a OR, odds ratio; CI, confidence interval.

Crude Model adjusted for none. Adjusted Model adjusted for all covariates.

### 3.4 Stratification analysis

**Figure 3:**
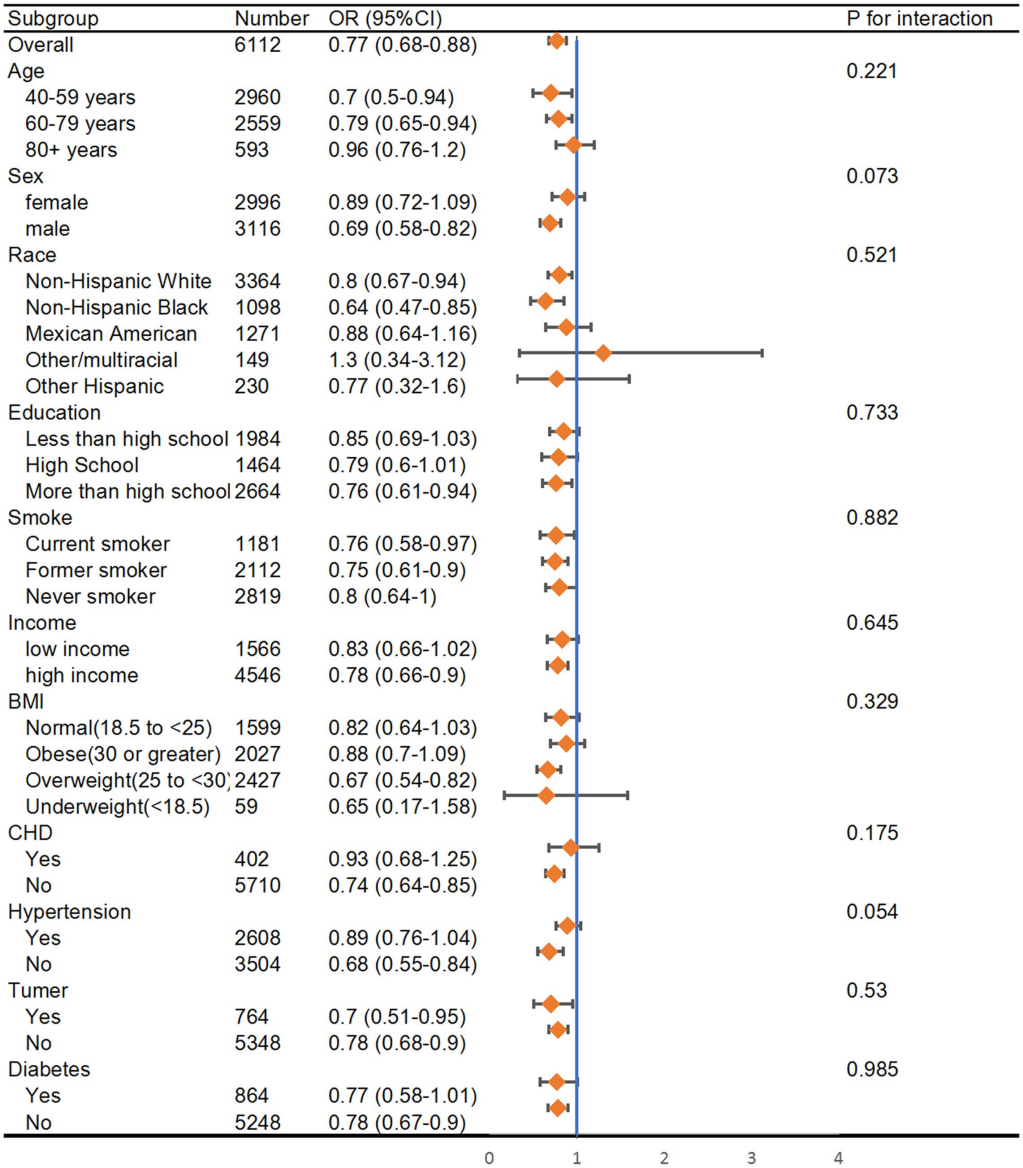
Subgroup analysis of the association between thiamine intake and risk of peripheral artery disease. The results were adjusted for all covariates except the corresponding stratification variable. HR, hazard ratio; CI, confidence interval.

Stratified analyses were further conducted to evaluate the association of dietary thiamine and the risk of PAD in various subgroups according to the categories of age, sex, income, education, smoking state, and disease state (Hypertension, Diabetes, CHD, and Tumor) (Figure 3). The thiamine intake was treated as a continuous variable. The statistically significant negative associations between thiamine intake and PAD were found in male participants (OR = 0.69, 95% CI: 0.58–0.82), participants under 80 years of age (OR = 0.7, 95% CI: 0.5–0.94 in 40–59 years group; OR = 0.79, 95% CI: 0.65–0.94 in 60–79 years group), and former smokers (OR = 0.75, 95% CI: 0.61–0.0.9).

## 4 Discussion

In this large-scale cross-sectional study in US adults, we first demonstrated that thiamine intake was independently associated with decreased risk for peripheral artery disease. It is worth mentioning that there was some evidence of a dose–response relationship; thiamine intake was related to risk for PAD in an L-shaped nonlinear manner (***p*** for nonlinearity = 0.082), with the curves being flatter when thiamine intake was 0.65 mg/day and the lower risk of PAD at 0.65–1.13 mg/day. In the context of the restricted cubic spline (RCS) model, a *p*-value greater than 0.05 indicates that the modeled data trend does not significantly differ from the real-world data, confirming the validity of the L-shaped association. This should not be seen as a non-significant result, but rather as an indication that the model closely reflects the observed relationship. Further exploratory subgroup analysis showed no significant interactions.

There are several possible mechanisms to explain the link between lower levels of thiamine and increased risk of PAD. Firstly, thiamine and its derivative benfotiamine can serve as coenzymes in the central metabolic processes of glucose in the body (8). Reports indicate that thiamine can lower blood glucose levels (21). Thiamine-like drugs can reduce the damage to vascular endothelial cells caused by hyperglycemia (22, 23) and decrease apoptosis of vascular endothelial cells and surrounding cells (24). Studies have shown that thiamine protects human infra-genicular arterial smooth muscle cells from glucose and insulin-mediated proliferation (25), crucial for forming atherosclerotic plaques. Secondly, thiamine may protect vascular health by improving endothelial function (26, 27) and exerting antioxidant (28) and anti-inflammatory effects (29). It has been reported that endothelial function can be enhanced in environments rich in thiamine (22). These findings suggest a potential association between adequate thiamine intake and a lower risk of PAD, which may be related to effects on peripheral vascular resistance. However, excessive thiamine intake may harm or pose potential health risks (30). Consequently, a recent meta-analysis (31) suggested that solely administering thiamine supplements to critically ill patients could raise the likelihood of mortality. Additionally, there have been reports indicating that thiamine supplementation may potentially encourage the growth of tumors (32). Research results suggest that PAD risk increases when dietary thiamine intake exceeds 1.13 mg/day. Therefore, avoiding excessive thiamine intake and maintaining an appropriate intake level is advisable.

Moreover, alcohol consumption may also play a role in modulating the risk of PAD and instances of alcohol misuse are gradually increasing (33). Studies have shown a complex relationship between alcohol and PAD, where moderate alcohol consumption might have protective cardiovascular effects, while excessive alcohol intake significantly increases PAD risk (34). Chronic alcohol use is linked to thiamine deficiency (35), and thiamine supplementation is often recommended to prevent alcohol-related neurological complications like Wernicke-Korsakoff syndrome (36, 37). Given this interaction, it is possible that chronic alcohol users who receive high doses of thiamine supplementation might influence the observed association between thiamine intake and PAD risk. While our study did not include alcohol consumption as a variable, future research should consider the potential confounding effects of alcohol intake, especially in relation to thiamine supplementation and PAD risk. Addressing this interaction may provide further insights into the L-shaped association observed between thiamine intake and PAD risk.

Lastly, based on our research findings, we emphasize the importance of considering PAD risk from a nutritional perspective. Maintaining an appropriate intake of thiamine can lower PAD risk, especially for older individuals and women. Therefore, in clinical practice, attention should be paid to thiamine levels in these populations and ensure they reach appropriate levels.

In conclusion, moderate thiamine intake is closely associated with reducing PAD risk, while excessive intake may pose potential hazards. We underscore the importance of considering PAD risk from a nutritional standpoint and recommend focusing on thiamine intake levels for specific populations in practice. Future research can further explore the mechanisms of thiamine in PAD prevention to ensure our findings are more fully supported.

Strengths of the present study include the use of a territory-wide, well-validated electronic healthcare database with records of all diagnoses, hospitalizations, and details of drug dispenses. This database allows for collecting the relevant information required to preclude common biases in conventional observational studies, such as selection and recall biases. Secondly, by adjusting for socioeconomic status, comorbidities, and other potential confounding factors, we can enhance the validity of the conclusions.

There are several limitations in the present study. First, as an observational study, we cannot establish a causal relationship between thiamine intake and the risk of PAD in the US population. We need longitudinal studies with large sample sizes to confirm our results. Secondly, NHANES does not provide information on blood thiamine concentrations. Therefore, we cannot study the relationship between blood thiamine and PAD risk. However, the human body can only store a small amount of thiamine, with low absorption efficiency, and blood thiamine levels represent only 0.8% of total thiamine in the body (7). Thus, serum thiamine is not a reliable indicator of overall thiamine stores. Thirdly, we cannot conclusively generalize our results to other populations with different demographic data, as all participants were U.S. residents. Fourthly, we did not perform a multivariate analysis including alcohol consumption as a variable. Alcohol intake may potentially confounding the relationship between thiamine intake and PAD risk.

## 5 Conclusion

After multivariable adjustment, lower thiamine intake levels were significantly and nonlinearly associated with higher risks of PAD in American adults. We first discovered L-shaped associations between thiamine intake and the risk of PAD, with an inflection point at 0.66 mg/day and a lower risk of PAD at 0.76–1.13mg/day of dietary thiamine intake. These results should be considered when developing clinical guidelines and updates.

## Data Availability

Publicly available datasets were analyzed in this study. This data can be found here: https://wwwn.cdc.gov/nchs/nhanes/.

## Footnotes

^1^ http://www.cdc.gov/nchs/nhanes.htm

^2^ www.cdc.gov/nchs/nhanes/
